# Association Between Sociodemographic Variables, Healthy Habits, and Stress with Risk Scales for Liver Disease Associated with Metabolic Dysfunction

**DOI:** 10.3390/life15010116

**Published:** 2025-01-16

**Authors:** Ángel Arturo López-González, Emilio Martínez-Almoyna Rifá, Hernán Paublini Oliveira, Cristina Martorell Sánchez, Pedro Juan Tárraga López, José Ignacio Ramírez-Manent

**Affiliations:** 1 ADEMA-Health Group of University Institute of Health Sciences (IUNICS) of Balearic Islands, 07120 Palma, Spain; angarturo@gmail.com (Á.A.L.-G.); emilio@udemax.com (E.M.-A.R.); h.paublini@eue.edu.es (H.P.O.); c.martorell@eua.edu.es (C.M.S.); joseignacio.ramirez@ibsalut.es (J.I.R.-M.); 2 Faculty of Odontology, University School ADEMA-UIB, 07009 Palma, Spain; 3 Health Service of the Balearic Islands, 07003 Palma, Spain; 4 Faculty of Medicine, Castilla la Mancha University, 02071 Albacete, Spain; 5 Faculty of Medicine, Balearic Islands University, 07122 Palma, Spain

**Keywords:** metabolic dysfunction-associated fatty liver disease (MAFLD), nonalcoholic steatohepatitis (NASH), sociodemographic variables, Mediterranean diet, physical activity, stress

## Abstract

Metabolic dysfunction-associated fatty liver disease (MAFLD) is the most common cause of chronic liver disease worldwide, with a multifactorial etiology. This study aims to evaluate the associations between various sociodemographic variables, healthy habits, and stress with risk scale values for MAFLD. Materials and Methods: A descriptive, cross-sectional study was conducted on 16,708 Spanish workers to assess how sociodemographic variables (age, gender, and socioeconomic status), healthy habits (smoking, Mediterranean diet adherence, and physical activity), and stress correlate with values from three MAFLD risk scales: fatty liver index (FLI), hepatic steatosis index (HSI), and lipid accumulation product (LAP). Results: All analyzed variables were associated with the values of the three MAFLD risk scales. Among them, the variables showing the strongest associations (represented by odds ratio values) were age and physical activity. Conclusions: The profile of an individual at higher risk of elevated MAFLD risk scale values is a male, aged 50 or older, belonging to lower socioeconomic levels (manual laborers), a smoker, sedentary, with low adherence to the Mediterranean diet, and with high stress scale scores.

## 1. Introduction

Metabolic dysfunction-associated liver disease (MAFLD) represents a growing challenge for global public health due to its high prevalence. According to NHANES 2017-18 data, the weighted prevalence of non-MAFLD, MAFLD without fibrosis, and MAFLD with fibrosis was 47.05%, 36.67%, and 16.28%, respectively [1]. This condition is characterized by excessive fat accumulation in the liver in individuals without significant alcohol consumption, often linked to metabolic alterations such as obesity, insulin resistance, type 2 diabetes, and dyslipidemia [2]. In recent years, the transition to the term “metabolic dysfunction-associated liver disease” reflects a better understanding of its multifactorial etiology and close association with metabolic syndrome.

MAFLD is not only a marker of systemic metabolic dysfunction but also has the potential to progress to more severe liver diseases, including nonalcoholic steatohepatitis (NASH) [3], advanced fibrosis [4], cirrhosis, and hepatocellular carcinoma [5]. Furthermore, it is associated with an increased risk of cardiovascular disease, which is the leading cause of mortality in these patients [6].

MAFLD is defined as the presence of hepatic steatosis in more than 5% of hepatocytes, confirmed through histological studies or imaging techniques, in the absence of other secondary causes of liver damage, such as excessive alcohol consumption, chronic viral infections, or autoimmune diseases. This terminological shift emphasizes its systemic nature, as fat accumulation in the liver is not an isolated event but rather a manifestation of an underlying metabolic imbalance [7].

The spectrum of MAFLD ranges from simple hepatic steatosis, which is typically asymptomatic and reversible, to NASH, characterized by liver inflammation and cellular damage that, in some cases, leads to progressive fibrosis. This variability in severity highlights the importance of identifying patients at risk of progression to intervene early.

The global prevalence of MAFLD has alarmingly increased over the past decades, paralleling the epidemics of obesity and type 2 diabetes. It is estimated to affect 25% of the global population, with prevalence varying by region. In developed countries like the United States and Europe, rates reach 30–40%, while in regions such as Asia and the Middle East, rates range between 15% and 30% [8].

The impact of MAFLD is particularly high in certain population groups. For instance, individuals with obesity have a MAFLD prevalence exceeding 70% [9], and in those with type 2 diabetes, the figure may reach 80% [10]. The relationship between obesity and MAFLD could be explained by the fact that obesity and insulin resistance are endocrinopathies that may result solely from excessive nutritional intake and glycemic load, leading to increased insulin production and decreased peripheral sensitivity in adipose and muscle tissue. Obesity arises from a prolonged positive energy balance, which results in the storage of calories, primarily as triglycerides, in adipose tissue. When excess calories exceed the storage capacity of adipose tissue, triglycerides may accumulate ectopically, such as in the liver [11]. Studies have also identified significant ethnic disparities, with higher prevalence observed in Hispanic populations, followed by Caucasian and Asian populations, and a lower incidence among individuals of African descent [12].

Population aging and the increase in metabolic risk factors predict a continuous growth in the burden of MAFLD [13]. Additionally, due to the strong correlation between liver disease and cardiovascular diseases, a concomitant rise in mortality related to these conditions is projected.

MAFLD is commonly asymptomatic in its early stages, complicating early diagnosis. Most patients with simple steatosis exhibit no noticeable symptoms and are incidentally diagnosed through laboratory tests or imaging studies performed for other reasons. In more advanced cases, patients may experience fatigue, abdominal discomfort in the right upper quadrant, or signs of liver complications such as jaundice or ascites. However, these symptoms are non-specific and are typically associated with advanced stages of the disease, such as cirrhosis or hepatocellular carcinoma [14].

Additionally, MAFLD has significant extrahepatic manifestations. Insulin resistance, dyslipidemia, and low-grade chronic inflammation contribute to an increased risk of cardiovascular disease, the leading cause of mortality in this population. Associations with chronic kidney disease, obstructive sleep apnea, and endocrine disorders such as hypothyroidism and polycystic ovary syndrome have also been documented. These manifestations underscore the importance of a multidisciplinary approach in managing these patients [15].

The diagnosis of MAFLD requires a combined approach, including clinical evaluation, laboratory tests, imaging techniques, and, in some cases, liver biopsy.

Liver biopsy is the gold standard for diagnosing and assessing the severity of MAFLD. It allows differentiation between simple steatosis and NASH and quantification of liver fibrosis. Nevertheless, the invasive nature, high cost, sampling variability, associated complications, and impracticality of liver biopsy for large-scale screening have underscored the need for non-invasive approaches to early diagnosis and staging. Over the past decade, numerous non-invasive serum biomarkers and scoring systems have been developed; however, none have yet proven capable of fully replacing liver biopsy. There remains a critical need for accurate and reliable non-invasive methods to enable the early detection and evaluation of steatohepatitis and fibrosis, facilitating timely risk stratification and intervention to prevent disease progression and associated complications [16].

Techniques such as ultrasound elastography or magnetic resonance imaging (MRI) [17] enable the measurement of liver stiffness, an indirect marker of fibrosis. MRI with fat quantification techniques, such as proton density fat fraction (PDFF), has demonstrated high accuracy in detecting steatosis [18].

The aim of this study is to evaluate the association between sociodemographic variables (age, gender, and socioeconomic status), healthy habits (smoking, adherence to the Mediterranean diet, and physical activity), and stress with MAFLD risk scale values in a working population without habitual alcohol consumption. The authors propose this objective because no study has been found in the reviewed literature that simultaneously evaluates this set of variables.

## 2. Materials and Methods

### 2.1. Participants

This study was based on observational research with a cross-sectional and descriptive design, including 16,708 workers from various employment sectors across different regions of Spain. The sample comprised 7556 men (45.2%) and 9152 women (54.8%). Participants were selected from individuals undergoing mandatory annual medical check-ups provided by their employers during the study period. Data collection took place between January 2019 and June 2020.

The inclusion criteria are as follows:

- Aged between 18 and 69 years.
- Employed in one of the participating companies.
- Provided consent to participate in the research.
- Authorized the use of their data for epidemiological purposes.
- Not habitual alcohol consumers.

The exclusion criteria are as follows:

- Age under 18 years or over 69 years.
- No employment contract with a participating company.
- Did not provide informed consent to participate in the study.
- Did not authorize the use of their data for epidemiological purposes.
- Habitual alcohol consumers.

**Figure 1:**
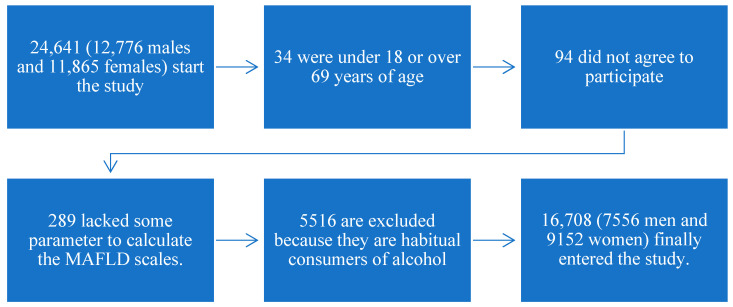
Flowchart for worker inclusion in the study.

Figure 1 illustrates the flowchart for worker inclusion in the study.

### 2.2. Determination of Variables

The occupational health teams of the collaborating companies were responsible for gathering information through the following methodologies:

- Clinical History: Sociodemographic data (age, sex, and occupation) and health-related aspects such as tobacco use, physical activity, adherence to the Mediterranean diet, and stress levels were collected.
- Physical and Clinical Measurements: Height, weight, waist and hip circumference, as well as systolic and diastolic blood pressure, were recorded.
- Laboratory Tests: Parameters such as lipid profile, liver function, and blood glucose levels were analyzed.

#### 2.2.1. Anthropometric Determinations

To minimize potential biases, standardized protocols were followed for all measurements. Height and weight were measured using a SECA 700 scale and a SECA 220 stadiometer (SECA, Chino, CA, USA), with participants in their underwear. Waist circumference was determined using a SECA measuring tape (SECA, Chino, CA, USA), taking measurements between the last rib and the iliac crest, while hip circumference was recorded at the widest point of the buttocks, with the individual relaxed and standing upright.

#### 2.2.2. Clinical Determinations

Blood pressure was measured using an OMRON-M3 sphygmomanometer (OMRON, Osaka, Japan) after 10 min of seated rest, avoiding food, beverage, or tobacco consumption for at least one hour prior. Three measurements were taken at one-minute intervals, and the average was calculated.

#### 2.2.3. Analytical Determinations

Blood samples were drawn via venipuncture after 12 h of fasting, refrigerated, and processed in reference laboratories within 72 h. Analyses included enzymatic methods for triglycerides, total cholesterol, and glucose. HDL cholesterol was measured by precipitation, and LDL cholesterol was calculated using the Friedewald formula when triglycerides were below 400 mg/dL [19]. When triglycerides were higher than 400 mg/dL, LDL-c was obtained by direct estimation.

#### 2.2.4. Risk Scales

The following metabolic dysfunction-associated liver disease (MAFLD) risk scores were calculated:

- Fatty liver index (FLI) [20]:

FLI = e^0.953 × log(triglycerides) + 0.139 × BMI + 0.718 × log(GGT) + 0.053 × waist circumference − 15.7451^ + e^0.953 × log(triglycerides) + 0.139 × BMI + 0.718 × log(GGT) + 0.053 × waist circumference − 15.745^ × 100.

Scores ≥ 60 were considered high risk of MAFLD.

- Hepatic steatosis index (HSI) [21]:

HSI = 8 × (AST/ALT) + BMI + 2 (if diabetic) + 2 (if female)HSI Scores ≥ 36 were considered high risk of MAFLD.

- Lipid accumulation product (LAP) [22]:
  ○ Men: (waist circumference (cm) − 65) × triglycerides (mMol).
  ○ Women: (waist circumference (cm) − 58) × triglycerides (mMol).

Scores ≥ 42.7 were considered high risk of MAFLD.

### 2.3. Operational Definitions

- Occupational Category: Classified according to the Spanish Society of Epidemiology, based on the National Occupations Classification 2011. Manual workers (blue collar) included operators and technicians, while non-manual workers (white collar) consisted of executives and university professionals [23].
- Tobacco Use: Defined as smoking at least one cigarette daily within the past 30 days or having quit less than one year prior.
- Adherence to the Mediterranean Diet: Assessed using the PREDIMED questionnaire, with high adherence defined as a score of 9 or higher [24]. (Questionnaire at the end of the article)
- Physical Activity: Measured using the International Physical Activity Questionnaire (IPAQ), which accounts for the frequency, duration, and intensity of exercise [25]. (Questionnaire at the end of the article)
- Stress: Evaluated using Cohen’s Perceived Stress Scale (PSS), an internationally validated tool [26].

### 2.4. Statistical Analysis

Descriptive analysis of categorical variables was performed using frequencies and distributions. The Kolmogorov–Smirnov test was applied to assess the normality of quantitative variables, followed by the calculation of means and standard deviations. In the bivariate analysis, Student’s *t*-test was used to compare means, while the chi-square test evaluated proportions. Variables related to MAFLD were examined using a multinomial logistic regression model, with goodness-of-fit assessed through the Hosmer-Lemeshow test. A stratified analysis was conducted to identify potential confounding factors, although none of the variables analyzed exhibited such effects. Statistical processing was performed using SPSS software version 29.0 (Licensed Material. Property of IBM Corp © Copyright IBM Corporation (Armonk, NY, USA) and its licensors 1989, 2021), with a significance level of 0.05.

## 3. Results

The anthropometric and clinical results of the 16,708 participants are summarized in Table 1. The mean age was just over 44 years, with a higher proportion in the 40–69 age range. Men exhibited worse anthropometric, clinical, and analytical indicators than women. Approximately 27% of participants were smokers, and nearly 50% engaged in regular physical activity. Statistically significant gender differences were observed in all cases (*p* < 0.001).

Table 2 and Table 3 present the mean values and the prevalence of high-risk scores for MAFLD scales according to sociodemographic variables, healthy habits, and stress levels. These values increase with age and are higher among manual workers, smokers, sedentary individuals, those with low adherence to the Mediterranean diet, and individuals with high stress levels. In all cases, the values are lower in women. All differences show high statistical significance (*p* < 0.001).

Table 4 presents the results of the multinomial logistic regression. All analyzed variables are associated with the MAFLD risk scale values. Among them, the variables showing the strongest associations (highest odds ratio values) are age and physical activity. In all cases, the observed differences demonstrate high statistical significance (*p* < 0.001).

## 4. Discussion

In our study, all analyzed variables were associated with elevated MAFLD risk values. Understanding how variables such as age, gender, socioeconomic status, lifestyle habits, and stress affect MAFLD is critical for designing personalized prevention, diagnosis, and treatment strategies.

Age is a crucial determinant in the prevalence and severity of MAFLD, with its impact manifesting differently across various life stages. Epidemiological studies have shown that this disease is more prevalent among middle-aged and older adults, peaking during the fifth and sixth decades of life [27]. This finding aligns with the results of our study, underscoring the importance of considering age as a key risk factor.

The progressive increase in adverse metabolic factors such as obesity, insulin resistance, and type 2 diabetes, which tend to become more prevalent with aging, may explain this trend. Additionally, age-related changes, such as the redistribution of body fat toward greater visceral adiposity and reduced physical activity, significantly contribute to the development of MAFLD.

However, it is important to emphasize that this condition is not confined to older adults. Recent studies have identified cases of MAFLD in adolescents, particularly those with obesity or metabolic syndrome, suggesting that the accumulation of metabolic risks may begin much earlier than previously thought. These findings highlight the need for prevention and management strategies that span all life stages, from childhood to old age, to mitigate the impact of MAFLD [28].

Sex is a determinant factor in the epidemiology, clinical presentation, and progression of MAFLD, as confirmed by the results of our study. The disease is generally more prevalent in men than in premenopausal women, which may be attributed to the protective effects of estrogen on liver metabolism, visceral fat accumulation, and insulin sensitivity [29]. However, this biological advantage diminishes significantly after menopause, when estrogen levels drop sharply. At this stage of life, women not only match but, in some cases, exceed the prevalence rates of MAFLD observed in men [30].

These differences also influence the nature of associated comorbidities. Men with MAFLD tend to have a higher risk of developing cardiovascular disease due to a more adverse metabolic profile. In contrast, postmenopausal women often experience more severe metabolic complications, such as pronounced insulin resistance, adipose tissue dysfunction, and greater ectopic fat accumulation. These findings highlight the need for sex-specific approaches in the prevention, diagnosis, and therapeutic management of MAFLD to address the unique needs of each group and maximize the effectiveness of interventions [31].

Socioeconomic status (SES) is deeply linked to MAFLD prevalence and management, as reflected in our findings. Individuals with lower SES are more likely to experience higher rates of obesity [32] and metabolic syndrome [33], key risk factors for MAFLD. This is partly due to limited access to healthy foods, physical activity opportunities, and quality medical education [34]. Furthermore, dietary patterns in low-SES populations often feature ultraprocessed foods rich in trans fats, refined carbohydrates, and sugars, all of which promote hepatic steatosis. Limited access to mental health resources can also exacerbate the impact of chronic stress, increasingly recognized as a significant contributor to MAFLD development [35]. In contrast, individuals with higher SES are more likely to adopt healthy behaviors such as a balanced diet and regular physical activity, which can protect against MAFLD. Addressing socioeconomic inequalities is therefore a priority in effectively tackling this disease [36,37].

Tobacco consumption is a well-established risk factor for various chronic diseases, including cardiovascular and respiratory conditions, and its relationship with MAFLD has garnered increasing attention in the scientific community. In our study, we identified a significant association between smoking and MAFLD, highlighting how this habit contributes to the development and progression of the disease. Smoking exacerbates liver damage through multiple mechanisms, including oxidative stress, chronic inflammation, and insulin resistance. These alterations not only promote fat accumulation in the liver but also contribute to the progression of steatosis to more severe stages, such as NASH and hepatic fibrosis. Additionally, smokers tend to exhibit elevated triglyceride levels and reduced HDL cholesterol [38], which worsens the metabolic risk associated with MAFLD. Nicotine, one of the most studied components of tobacco, stimulates hormones such as cortisol and catecholamines, increasing lipolysis and elevating plasma free fatty acid levels. Former smokers also show an increased predisposition, suggesting a lasting relationship between smoking and lipid alterations [39]. These free fatty acids not only impair beta-cell function, contributing to glucose intolerance and insulin resistance, but also accumulate in the liver, exacerbating hepatic damage. Recent studies indicate that smokers have a significantly higher risk of progressing from simple steatosis to NASH and advanced fibrosis, even when other metabolic factors are controlled [40]. This finding underscores the independent role of smoking in the pathogenesis of MAFLD. Furthermore, smoking has been observed to increase the risk of hepatocellular carcinoma in patients with MAFLD, likely through the activation of specific oncogenic pathways in the liver [41]. Finally, the impact of passive smoking should not be overlooked, especially in children and adolescents, who are particularly susceptible to developing adverse metabolic conditions. These results emphasize the importance of incorporating smoking cessation interventions and protecting vulnerable populations into MAFLD management programs [42].

Adherence to the Mediterranean diet (MD) is recognized as an effective strategy not only for preventing and managing MAFLD but also for improving overall metabolic health, supporting the findings of our study. This dietary pattern, characterized by a high intake of healthy foods such as fruits, vegetables, whole grains, olive oil, fish, and nuts, alongside limited consumption of red meat, refined sugars, and trans fats, provides multiple metabolic benefits. The MD enhances insulin sensitivity and reduces systemic inflammation, two key factors in the pathogenesis of MAFLD. Additionally, it directly impacts liver health by decreasing hepatic lipid accumulation and attenuating fibrosis, an early marker of progressive liver damage [43]. Systematic reviews and meta-analyses have shown that greater adherence to the MD, due to its abundance of antioxidants, monounsaturated fatty acids, and polyphenols, is significantly associated with a lower prevalence of MAFLD and a reduced likelihood of progression to advanced forms of the disease, such as NASH or advanced fibrosis [44]. Individual components of the MD, such as olive oil, provide healthy fats, while polyphenols in fruits and vegetables possess hepatoprotective properties [45]. In our study, we comprehensively assessed adherence to the MD and its relationship with the risk of developing MAFLD. The results revealed a robust association between non-adherence to this dietary pattern and an increased risk of MAFLD, with odds ratios (OR) ranging from 1.76 to 1.89 across the three indices evaluated. These findings highlight the importance of promoting this dietary pattern as a key preventive measure, particularly among high-risk populations. Furthermore, we identified non-adherence to the MD as the second most influential modifiable risk factor for developing MAFLD, underscoring the need to strengthen public policies aimed at encouraging this dietary pattern.

Physical activity performed regularly stands out in our study as the modifiable risk factor with the greatest protective effect against the risk of MAFLD. In our study, individuals who do not engage in regular exercise show a significantly higher prevalence of the risk of developing MAFLD in both sexes. Multinomial logistic regression analyses confirm that physical inactivity is the most influential modifiable risk factor for developing MAFLD, with an OR ranging from 2.12 to 2.60. These findings highlight the importance of addressing physical inactivity as a priority in prevention and management strategies. Both aerobic exercise and resistance training have been shown to improve metabolic markers and reduce hepatic fat accumulation, even without significant weight loss [46]. These benefits go beyond simple weight reduction, including enhanced insulin sensitivity, reduced systemic inflammation, and increased hepatic fatty acid oxidation [47]. Furthermore, studies suggest that exercise not only prevents the onset of MAFLD but may also delay the progression of fibrosis in patients with NASH [48]. In this context, our results emphasize the importance of promoting active lifestyles as a key strategy in the prevention of MAFLD, particularly in populations more vulnerable to sedentary behavior and its associated consequences. Incorporating regular physical activity into daily life is crucial to reduce the risk of liver disease and improve overall metabolic health.

Stress, as observed in our research, is associated with an increased risk of MAFLD, highlighting the importance of considering it as a risk factor that is often overlooked in previous studies. In our analysis, the results show an odds ratio (OR) ranging from 1.52 to 1.92, placing it at a similar level to other key factors, such as lack of adherence to the Mediterranean diet. This finding underscores the need to integrate stress management into preventive and therapeutic strategies for MAFLD, given its potential impact on liver health. Chronic stress has emerged as a significant risk factor in the progression of this disease. Recent research suggests that psychological stress contributes to the accumulation of liver fat and promotes progression to more severe forms of the disease through the activation of the hypothalamic–pituitary–adrenal axis, resulting in the sustained release of glucocorticoids. These glucocorticoids promote hepatic lipogenesis, favoring the accumulation of fat in the liver, and contribute to insulin resistance, another critical factor in the development of MAFLD. Additionally, a high prevalence of stress has been identified among patients with MAFLD, particularly those with psychiatric comorbidities, such as depression and anxiety [49]. Therefore, integrating stress management strategies, such as mindfulness interventions, psychological support, and relaxation techniques, could be a fundamental component in a comprehensive approach to treat and prevent MAFLD, improving both the physical and emotional health of patients.

The aging population and the increase in metabolic risk factors predict a continuous growth in the burden of MAFLD. Over the years, this disease is associated with an increasing number of comorbidities, such as chronic kidney disease, obstructive sleep apnea, and endocrine disorders like hypothyroidism and polycystic ovary syndrome. These coexisting conditions worsen the patient’s prognosis, highlighting the importance of a multidisciplinary approach in managing these patients, integrating various medical specialties to improve treatment and follow-up of this complex pathology. The variability in the severity of MAFLD also underscores the need to identify patients at risk of progression early, which will allow timely interventions and prevent severe complications. Liver biopsy, although considered the gold standard for diagnosing and assessing the severity of NASH, has limitations, as it is an invasive technique unsuitable for population screening. Instead, non-invasive techniques such as ultrasound elastography or magnetic resonance imaging can be useful for measuring liver stiffness, an indirect marker of fibrosis. However, these methods also have limitations, such as their high cost, restricting their utility in large-scale screening programs. Currently, the global population is progressively aging, leading to an increase in public spending to address the growing social and healthcare needs of older adults. One of the most important determinants of healthcare costs is the general health status of the population. To address this increasing demand, it is crucial to implement effective public health strategies. Establishing healthcare policies focused on the prevention and management of modifiable risk factors, such as health education at all socioeconomic levels, improving healthy lifestyle habits, and paying special attention to mental health, are key measures. Implementing preventive activities from the younger stages of life will not only improve the population’s health but also contribute to reducing healthcare costs while enhancing the overall quality of life. Additionally, these strategies have the potential to reduce long-term economic burdens by preventing costly chronic diseases and improving healthcare system efficiency.

The importance of this study lies in reinforcing the utility of MAFLD risk scales as effective and cost-effective screening tools for early detection of this condition, especially in high-risk populations. By highlighting the relevance of these scales, the study advocates for the integration of these strategies within public health systems to optimize available resources and reduce costs associated with the treatment of advanced diseases. Additionally, it underscores the need to implement health education policies that effectively reach economically disadvantaged populations, who often face barriers to accessing information and preventive medical care. Moreover, it highlights the importance of mental health as an emerging risk factor in the development of MAFLD, implying a comprehensive approach that considers both metabolic and psychological factors in disease management. To address this aspect, it would be essential to increase resources dedicated to mental health within the public health system, which would improve its efficiency and, in the long term, prevent or reduce the incidence of MAFLD in the population.

## 5. Study Strengths and Limitations

Our study’s strengths include its large sample size (over 16,000 participants) and the wide range of analyzed variables, making it a reference study for assessing the association of sociodemographic variables, healthy habits, and stress with MAFLD.

Limitations include the descriptive nature of the study, which precludes establishing causal relationships, only associations.

Another limitation is that MAFLD was determined using validated risk scales rather than objective methods such as abdominal ultrasound or liver biopsy. Another potential limitation is that possible confounding factors, such as the presence of comorbidities or treatments that could affect the presence of MAFLD, were not considered due to the lack of available information.

## 6. Conclusions

MAFLD is a multifaceted condition influenced by a broad range of demographic, socioeconomic, and lifestyle factors. The evidence reviewed highlights the importance of adopting a comprehensive approach to address this disease, including promoting healthy behaviors, reducing social inequalities, and integrating psychological interventions.

Given MAFLD’s significant public health impact, further research is essential to explore how these factors interact, identify high-risk patient subgroups, and develop tailored prevention and treatment strategies. Additionally, the findings underscore the need to strengthen public health policies that promote healthy lifestyle habits, particularly in vulnerable populations.

With a multidisciplinary and equitable approach, it is possible to effectively address the growing burden of MAFLD and improve clinical outcomes in this population.

The PREDIMED questionnaire used is available in Supplementary Materials.

## Figures and Tables

**Table 1:** Characteristics of the population.

	Men *n* = 7556	Women *n* = 9152	
	Mean (SD)	Mean (SD)	*p*-Value
Age (years)	44.7 (8.6)	43.4 (8.6)	<0.001
Height (cm)	173.7 (6.7)	161.8 (5.9)	<0.001
Weight (kg)	81.6 (13.5)	64.9 (11.9)	<0.001
Waist circumference (cm)	94.3 (10.9)	86.47 (14.3)	<0.001
Hip circumference (cm)	103.3 (9.9)	101.7 (11.5)	<0.001
Systolic blood pressure (mmHg)	133.9 (18.4)	120.98 (16.4)	<0.001
Diastolic blood pressure (mmHg)	81.0 (12.3)	74.9 (10.8)	<0.001
Total cholesterol (mg/dL)	205.1 (40.1)	195.9 (35.3)	<0.001
HDL-cholesterol (mg/dL)	51.0 (11.3)	60.3 (12.8)	<0.001
LDL-cholesterol (mg/dL)	129.6 (51.3)	118.6 (31.3)	<0.001
Triglycerides (mg/dL)	127.9 (86.8)	85.7 (52.0)	<0.001
Glucose (mg/dL)	93.6 (21.0)	88.6 (15.7)	<0.001
AST (U/L)	27.9 (15.8)	17.9 (10.8)	<0.001
ALT (U/L)	25.6 (14.2)	18.1 (7.7)	<0.001
GGT (U/L)	30.9 (29.8)	19.3 (14.4)	<0.001
	%	%	*p*-value
<30 years	4.8	7.0	<0.001
30–39 years	22.9	23.2	
40–49 years	39.7	45.9	
50–69 years	32.6	23.9	
Blue collar	7.57	21.2	<0.001
White collar	92.4	78.8	
Non-smokers	71.7	74.5	<0.001
Smokers	28.3	25.5	
No physical activity	51.5	50.1	<0.001
Physical activity	48.5	49.9	
No Mediterranean diet	53.9	52.3	<0.001
Mediterranean diet	46.1	47.7	
No stress	86.1	87.6	<0.001
Stress	13.9	12.4	

HDL, high-density lipoprotein; LDL, low-density lipoprotein; AST, aspartate aminotransferase; ALT, alanine aminotransferase; GGT, gamma-glutamyl transferase.

**Table 2:** Mean values of nonalcoholic fatty liver disease according to sociodemographic variables, healthy habits, and stress by gender (Student’s *t*-test).

		FLI		HSI		LAP	
Men	*n*	Mean (SD)	*p*-Value	Mean (SD)	*p*-Value	Mean (SD)	*p*-Value
<30 years	364	25.8 (21.3)	<0.001	33.9 (6.9)	<0.001	22.8 (20.9)	<0.001
30–39 years	1728	34.8 (25.9)		37.2 (7.1)		31.5 (27.4)	
40–49 years	3000	42.8 (25.4)		38.1 (7.0)		37.0 (30.8)	
50–69 years	2464	44.7 (24.8)		38.8 (7.2)		37.8 (30.5)	
Blue collar	572	35.8 (24.4)	<0.001	35.6 (7.0)	<0.001	31.5 (28.6)	<0.001
White collar	6984	39.9 (26.6)		38.9 (7.3)		34.3 (30.1)	
Non-smokers	5420	36.3 (23.9)	<0.001	36.6 (7.2)	<0.001	32.1 (29.1)	<0.001
Smokers	2136	38.8 (25.1)		38.8 (7.4)		34.0 (28.8)	
No physical activity	3888	30.8 (24.1)	<0.001	33.3 (7.7)	<0.001	31.3 (27.8)	<0.001
Physical activity	3668	42.3 (23.8)		41.9 (7.9)		38.2 (25.6)	
No Mediterranean diet	4072	31.5 (25.1)	<0.001	34.6 (7.9)	<0.001	32.6 (28.0)	<0.001
Mediterranean diet	3484	40.3 (24.0)		40.5 (8.0)		37.5 (27.9)	
No stress	6504	35.6 (24.0)	<0.001	34.8 (7.7)	<0.001	32.0 (27.9)	<0.001
Stress	1052	39.9 (25.6)		40.3 (8.0)		38.9 (28.3)	
Women	*n*	Mean (SD)	*p*-value	Mean (SD)	*p*-value	Mean (SD)	*p*-value
<30 years	640	13.6 (18.8)	<0.001	33.2 (6.6)	<0.001	14.7 (14.9)	<0.001
30–39 years	2124	17.5 (21.9)		36.2 (7.2)		17.2 (16.8)	
40–49 years	4196	20.6 (22.9)		37.2 (6.8)		18.9 (17.3)	
50–69 years	2192	26.3 (23.3)		38.1 (6.9)		20.3 (20.1)	
Blue collar	1940	16.9 (20.3)	<0.001	31.3 (6.9)	<0.001	16.4 (15.3)	<0.001
White collar	7212	22.8 (19.9)		37.6 (7.3)		19.2 (18.3)	
Non-smokers	6820	17.9 (20.1)	<0.001	32.6 (7.0)	<0.001	16.9 (15.8)	<0.001
Smokers	2332	21.5 (18.6)		36.1 (7.3)		18.6 (16.1)	
No physical activity	4584	16.3 (19.6)	<0.001	30.7 (7.3)	<0.001	14.6 (14.8)	<0.001
Physical activity	4568	23.6 (20.1)		41.0 (8.1)		20.2 (15.5)	
No Mediterranean diet	4786	17.3 (20.0)	<0.001	32.2 (7.5)	<0.001	15.3 (15.0)	<0.001
Mediterranean diet	4366	22.2 (21.1)		40.2 (7.7)		19.1 (14.8)	
No stress	7926	17.6 (20.5)	<0.001	33.5 (7.6)	<0.001	16.3 (14.6)	<0.001
Stress	1226	21.9 (21.0)		39.5 (7.4)		18.6 (14.4)	

FLI, fatty liver index; HSI, hepatic steatosis index; LAP, lipid accumulation product.

**Table 3:** Prevalence of high values of nonalcoholic fatty liver disease according to sociodemographic variables, healthy habits, and stress by gender (chi-square test).

		FLI High		HSI High		LAP High	
Men	*n*	%	*p*-Value	%	*p*-Value	%	*p*-Value
<30 years	364	11.9	<0.001	32.1	<0.001	23.9	<0.001
30–39 years	1728	20.8		40.8		35.6	
40–49 years	3000	28.5		50.6		44.8	
50–69 years	2464	31.3		55.8		48.9	
Blue collar	572	21.5	<0.001	48.5	<0.001	38.3	<0.001
White collar	6984	25.8		52.3		41.6	
Non-smokers	5420	24.9	<0.001	48.1	<0.001	40.7	<0.001
Smokers	2136	25.6		49.9		41.5	
No physical activity	3888	20.6	<0.001	38.1	<0.001	33.5	<0.001
Physical activity	3668	29.9		51.9		46.3	
No Mediterranean diet	4072	21.3	<0.001	40.1	<0.001	35.3	<0.001
Mediterranean diet	3484	28.5		49.2		45.6	
No stress	6504	23.3	<0.001	43.3	<0.001	35.6	<0.001
Stress	1052	27.9		52.5		44.6	
Women	*n*	%	*p*-value	%	*p*-value	%	*p*-value
<30 years	640	6.1	<0.001	30.5	<0.001	19.6	<0.001
30–39 years	2124	8.2		40.1		23.3	
40–49 years	4196	8.8		46.8		28.9	
50–69 years	2192	12.1		58.3		38.8	
Blue collar	1940	4.5	<0.001	32.2	<0.001	18.9	<0.001
White collar	7212	9.1		45.6		29.9	
Non-smokers	6820	8.3	<0.001	43.5	<0.001	26.9	<0.001
Smokers	2332	8.9		45.0		29.5	
No physical activity	4584	5.3	<0.001	35.6	<0.001	20.3	<0.001
Physical activity	4568	10.9		47.5		38.5	
No Mediterranean diet	4786	6.1	<0.001	37.6	<0.001	22.6	<0.001
Mediterranean diet	4366	9.5		46.1		35.9	
No stress	7926	6.6	<0.001	38.6	<0.001	26.6	<0.001
Stress	1226	10.8		49.9		38.4	

FLI, fatty liver index; HSI, hepatic steatosis index; LAP, lipid accumulation product.

**Table 4:** Multinomial logistic regression.

	FLI High		HSI High		LAP High	
	OR (95% CI)	*p*-Value	OR (95% CI)	*p*-Value	OR (95% CI)	*p*-Value
Women	1		1		1	
Men	2.89 (2.60–3.19)	<0.001	1.65 (1.55–1.76)	<0.001	1.91 (1.71–2.12)	<0.001
<30 years	1		1		1	
30–39 years	1.23 (1.19–1.27)	<0.001	1.32 (1.24–1.40)	<0.001	1.33 (1.28–1.39)	<0.001
40–49 years	1.74 (1.62–1.87)	<0.001	1.85 (1.60–2.11)	<0.001	1.77 (1.60–1.95)	<0.001
50–69 years	2.60 (2.29–2.92)	<0.001	2.92 (2.59–3.26)	<0.001	2.48 (2.10–2.86)	<0.001
White collar	1		1		1	
Blue collar	1.48 (1.33–1.64)	<0.001	1.39 (1.30.1.49)	<0.001	1.44 (1.30–1.58)	<0.001
Non-smokers	1		1		1	
Smokers	1.35 (1.28–1.43)	<0.001	1.29 (1.23–1.35)	<0.001	1.30 (1.20–1.41)	<0.001
Physical activity	1		1		1	
No physical activity	2.60 (2.33–2.88)	<0.001	2.12 (1.88–2.37)	<0.001	2.33 (2.01–2.65)	<0.001
Mediterranean diet	1		1		1	
No Mediterranean diet	1.89 (1.70–2.09)	<0.001	1.76 (1.60–1.93)	<0.001	1.80 (1.59–2.02)	<0.001
No stress	1		1		1	
Stress	1.69 (1.51–1.87)	<0.001	1.52 (1.41–1.63)	<0.001	1.92 (1.66–2.19)	<0.001

FLI, fatty liver index; HSI, hepatic steatosis index; LAP, lipid accumulation product; OR, odds ratio.

## Data Availability

Data are not available due to ethical or privacy restrictions. This study data are stored in a database that complies with all security measures at ADEMA-Escuela Universitaria. The Data Protection Delegate is Ángel Arturo López González.

## Supplementary Materials

The following supporting information can be downloaded at: http://www.mdpi.com/article/10.3390/life15010116/s1, PREDIMED questionnaire 1 and International Physical Activity Questionnaire.
